# Supplementary material for: GWAS and Meta-QTL Analysis of Kernel Quality-Related Traits in Maize
Source: Plants (Basel). 2024 Sep 29;13(19):2730. doi: 10.3390/plants13192730 (PMC11479128; doi:10.3390/plants13192730)
Supplement: Supplementary file 1 [file plants-13-02730-s001.zip › supplementary figure.pdf]

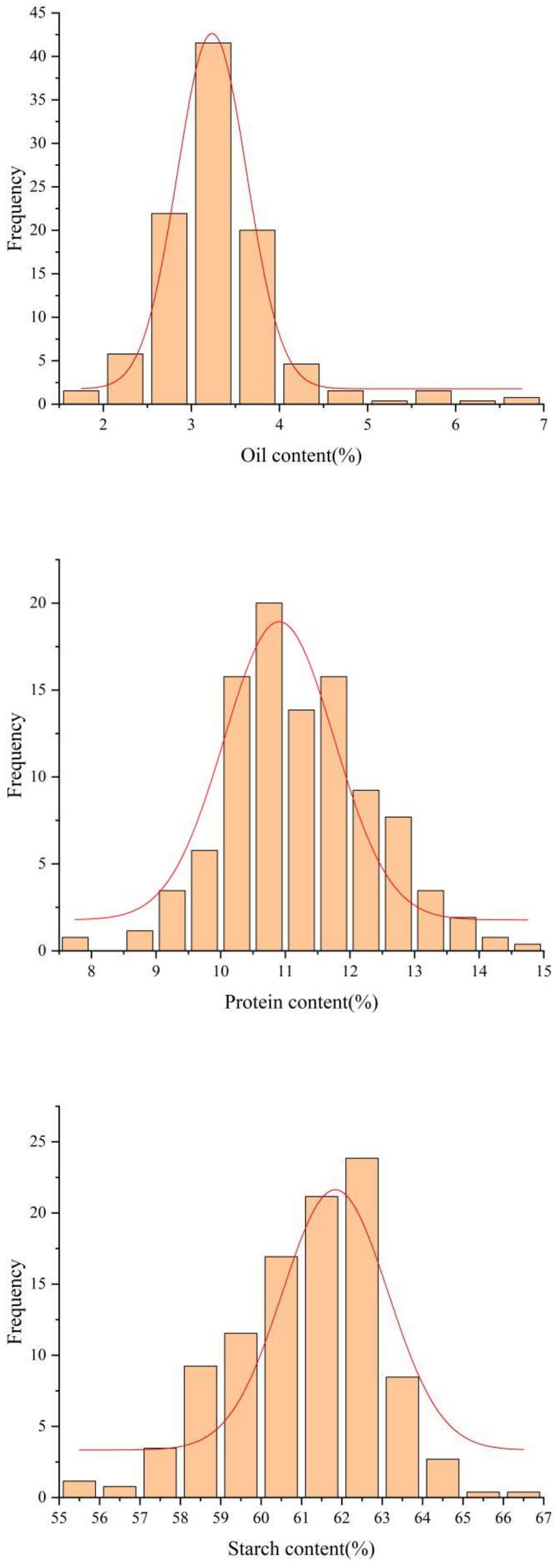

Figure S1. Frequency distribution histogram of protein content, oil content and starch content of maize inbred lines

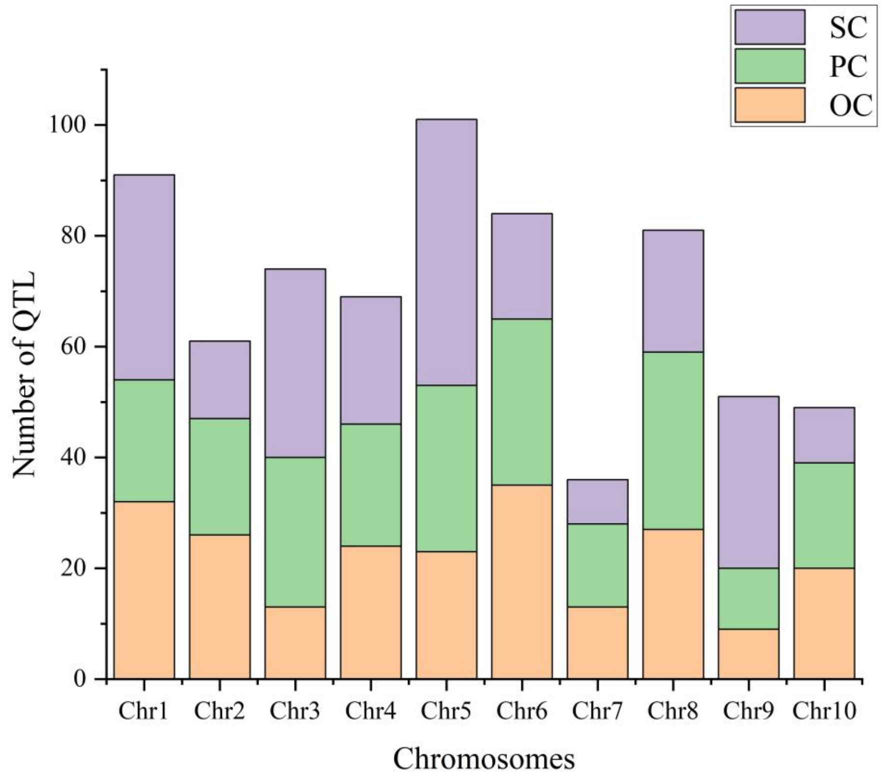

Figure S2. Initial QTL distribution on 10 chromosomes of quality traits. OC, oil content; PC,protein content; SC, starch content; Chr, chromosomes.

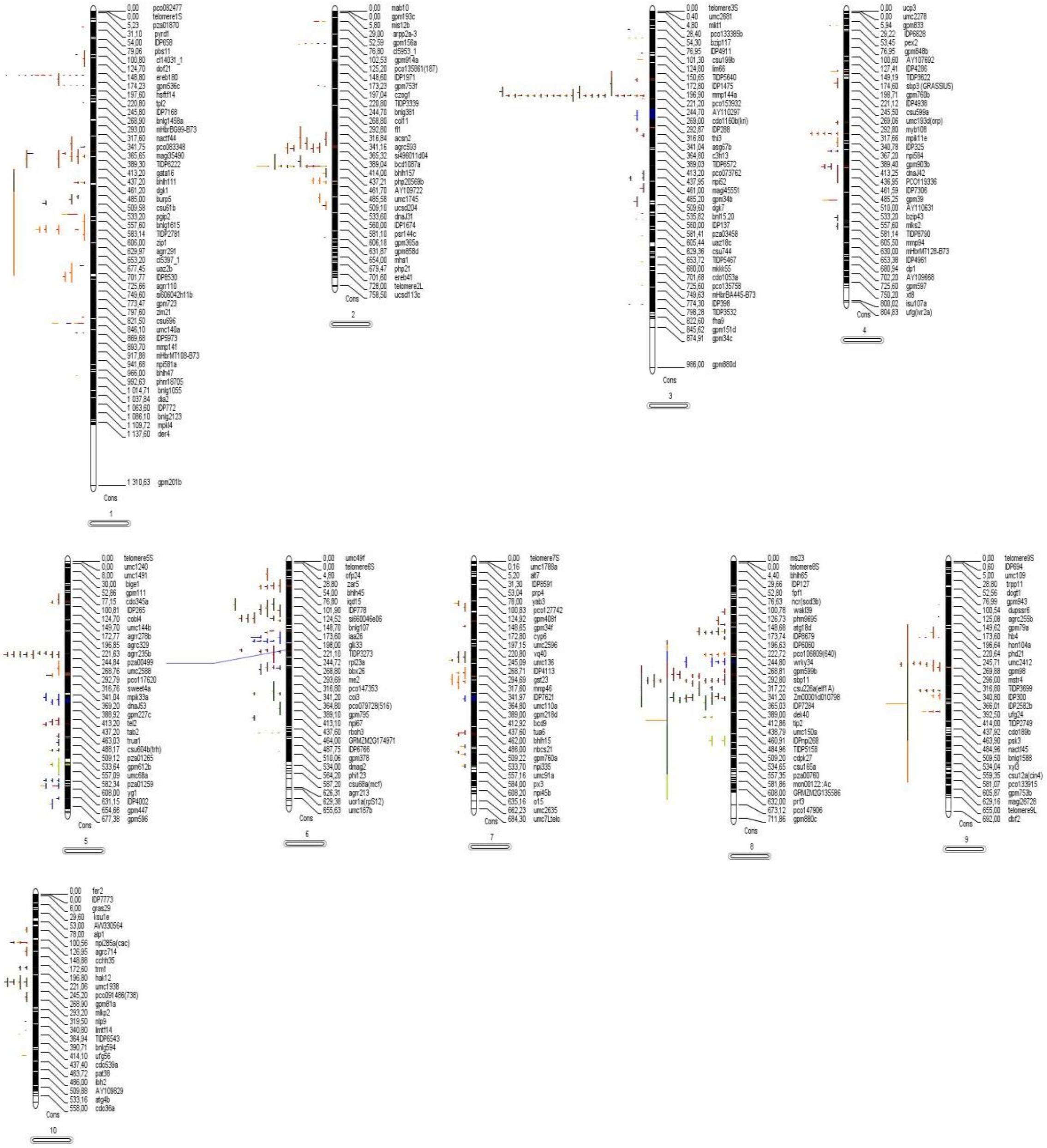

Figure S3. Projection and distribution of QTL and MQTL (Meta QTL) identified for quality traits. Bars on the left side of the chromosome correspond to QTL related to ear traits, black bars within chromosomes represent marker density, colored segments within the chromosome represent MQTL, on the right side of the chromosome are molecular markers and genetic distances (cM).
